# Poisonings in Poland reported to the Polish National Health Fund in the years 2009–2011

**DOI:** 10.1186/s40360-018-0254-x

**Published:** 2018-10-10

**Authors:** Aleksandra Świderska, Marek Wiśniewski, Marek Wiergowski, Anna Krakowiak, Jacek Sein Anand

**Affiliations:** 1 National Health Fund Headquarters, Warsaw, Poland; 2 Department of Clinical Toxicology, Medical University of Gdansk, Kartuska 4/6, 80-104, Gdansk, Poland; 3 Pomeranian Center of Toxicology, Gdansk, Poland; 4 Department of Forensic Medicine, Medical University of Gdansk, Gdansk, Poland; 5 Toxicology Department, Nofer Institute of Occupational Medicine, Lodz, Poland

**Keywords:** Poisoning, Epidemiology, Human toxicology, Poison control

## Abstract

**Background:**

Poisonings constitute a significant medical, social and economic problem worldwide. In Poland there is no nationwide registry of poisonings, which results in a lack of accurate epidemiological data. Few publications dealing with the problem are based on data obtained from toxicology units and therefore do not include information about cases treated at emergency departments and other non-toxicology units.

**Methods:**

We analyzed all admissions due to poisonings reported to the Polish National Health Fund by all hospital units in Poland in the 2009–2011 period. Diagnoses were encoded according to the ICD-10 classification.

**Results:**

A total of 254,425 admissions were reported, 85,398 in 2009, 85,230 in 2010 and 83,797 in 2011. The male to female ratios were 1.88, 1.75 and 1.80 respectively. The most frequent causes of admissions were poisonings with ethanol (*n* = 121,874; 47.9%), carbon monoxide (*n* = 17,179; 6.8%) and benzodiazepines (*n* = 10,340; 4.1%). Alcohols were the reason for 104,680 admissions in men (63.2%) and 22,612 admissions in women (25.5%; *p* < 0.01). Poisonings with pharmaceuticals and other drugs were reported in 34,616 men (20.9%) and 45,238 women (51%; *p* < 0.01). There were 1680 cases of fatal poisonings in the analyzed period. The hospital mortality due to poisonings increased from 1.1% in 2009 to 1.5% in 2011 (*p* < 0.01). The mortality in general Intensive Care Units increased from 14.4% in 2009 to 22.3% in 2011 (*p* < 0.01). The etiology of fatal poisonings was highly dependent on the type of hospital unit.

**Conclusions:**

The overall number of admissions due to poisonings decreased slightly during the study period, but they remained a significant cause of morbidity and mortality. Alcohols were the main cause of admissions in the analyzed period. Alcohol intoxications were more frequent in men while poisonings with pharmaceuticals were more frequent in women. Carbon monoxide exposures were a significant cause of morbidity and mortality in the studied period in Poland. A national poison information and toxicovigilance system should be created in Poland, ideally allowing for near real-time monitoring of cases of poisonings.

## Background

Poisonings constitute a significant medical, social and economic problem worldwide. Each case should be analyzed not only in the terms of associated morbidity and mortality, but also in the context of its influence on patient’s family members, relatives and the whole community [1–5].

The epidemiology of poisonings can be studied from different perspectives. These include overall mortality, hospital admission rates and enquiries to Poisons Information Services. Analyzing epidemiology of poisonings presents challenges depending on the source of data and the parameters analyzed. Analysis based on poison information calls is often an indication of exposures (very common in small children where there is usually only a suspicion of poisoning) and the analysis of cases treated in specialized toxicology unit causes a significant selection bias because they usually admit the only the most severe cases. Epidemiology varies with the geographical location. In developing countries poisoning by pesticides and herbicides is often common. In warmer countries people may be more often exposed to toxins from snakes or spiders. In most situations self-harm often involves agents that are available and may reflect local tradition. For example, in Sri Lanka pesticide and oleander poisoning are frequently encountered problems, in China herbicides and pesticides, and in the UK paracetamol are the most frequent poisons taken deliberately [6]. The epidemiology also changes with time, due to social trends, advances in medical care but also due to emerging new trends in substance abuse. The recent report from China shows decreasing mortality due to pesticide and alcohol poisonings [7]. Data from the United States shows increasing opioid mortality due to mostly abuse of heroin and synthetic opioids [8].

The system of poison control in Poland is based on Regional Centers of Clinical Toxicology (RCCTs) and was started in 1967. Currently, there are 11 RCCTs located in Gdansk, Poznan, Warsaw, Lodz, Lublin, Rzeszow, Tarnow, Wroclaw, Sosnowiec and Cracow (2 centers). All RCCTs in Poland are based on hospital units and include Intensive Care Units. The principal function of Polish RCCTs is the treatment of cases of poisonings which require specialist management (like antidote treatment or extracorporeal removal of poisons). The number of beds in the RCCTs is not big enough to treat all poisonings, so many less severe cases, which do not require intensive care, are treated at Emergency Departments (EDs) and other hospital units (mostly pediatric and internal medicine departments). Cases of severe poisonings which cannot be transferred to RCCTs are usually treated at general Intensive Care Units (ICUs). Since the creation of RCCTs there was no separate system of poison information. This service was provided by RCCTs without additional funding, usually by the physician on call at the RCCT. Until 2002 all Polish RCCTs sent annual reports of admissions to the RCCT in Lodz, which analyzed and published these data. The data on admissions to other units were never reported or analyzed. Until now there is no nationwide system of poison information or toxicovigilance in Poland. This situation results in a lack of accurate information on the morbidity and mortality of poisonings in Poland. Few published papers dealing with this problem are based or single-center experience or at best on the data obtained from all the RCCTs and do not include information about cases treated at EDs and other non-toxicology hospital units [4, 5, 9–13]. The most recent analysis of data from all the RCCTs in Poland covered years 1970–2002 and showed a decreasing overall mortality and an increasing number of admissions due to poisonings with alcohols [14]. The most recent analysis of mortality due to poisonings by Krakowiak et al. was based on data from only 5 of 11 active clinical toxicology centers in Poland [15]. Until now there has been no analysis of hospital admissions and mortality due to poisonings in Poland which included all types of hospital units.

The aim of this study was to analyze the epidemiology of poisonings based on the available data reported to the Polish National Health Fund (NHF) from 2009 to 2011.

## Methods

The study was approved by the Independent Bioethics Committee at Medical University in Gdansk (approval NKBBN/508/2017).

The healthcare system in Poland is mostly publicly funded, but a large number of providers (especially in outpatient care) are private entities. The funds are managed by the NHF, which reimburses the hospitals and outpatient clinics for the services performed for the people covered by the public health insurance. In the studied period the NHF covered around 80% of the Polish population.

The NHF database was created in 2003. The database is not public, permission to use it for the purpose of this study was granted by the president of the National Health Fund. The database is in an electronic form and contains millions of datasets reported annually by the Polish healthcare providers. The database is used to reimburse providers for healthcare services and to monitor and plan expenses of the NHF. In the 2009–2011 period the dataset reported by providers included: identifiers of the provider, patient’s personal identification number (contains age and sex information), time of admission and discharge, mode of admission and discharge, primary and secondary (optional) diagnoses (ICD-10), type of hospital unit and medical procedures performed (ICD-9). There was no regular quality control of the data reported by the healthcare providers to the NHF. The data used in this study were extracted from the NHF database. Data of all hospital admissions reported to the NHF in 2009–2011 were analyzed. We selected all cases in which primary diagnosis was within the T36-T65 range, according to the 10th revision of the International Statistical Classification of Diseases and Related Health Problems (ICD-10). The data on secondary diagnoses or intent of poisoning were not obligatorily reported to the NHF and because of it were only fragmentary (less than 20% of all reports) and were not used in this analysis. There were no other inclusion criteria, some incomplete records were excluded from the statistical analysis, as described in the results section.

Statistical analysis was performed using the Statistica 12 software (StatSoft, Inc., USA). Pearson’s chi-squared test was used for categorical variables and Student’s t-test was used for continuous data. All reported *p* values are two-sided, *p* values lower than the adopted level of significance (α = 0,05) were considered significant.

## Results

The number of hospitals (hospital units) which reported cases of poisonings to the NHF was 561 (2231) in 2009, 562 (1829) in 2010 and 553(2060) in 2011. The proportions of types of hospital units which reported cases of poisonings in the analyzed period are presented in Table 1.

**Table 1:** Hospital units reporting cases of poisonings to the NHF in the 2009–2011 period

Type of hospital unit	2009	2010	2011
Regional Center of Clinical Toxicology	9	9	11
Intensive Care Unit	246	254	310
Internal Medicine Unit	493	487	483
Pediatric Unit	352	356	353
Emergency Department	528	544	546
other	603	179	357

A total of 254,425 admissions of 240,517 patients with the diagnosis of poisonings were reported in the 2009–2011 period. The total number of reported admissions was higher than the total number of admitted patients because some patients were admitted due to poisonings more than once a year. The annual number of admissions decreased slightly throughout the analyzed period. There were 85,398 admissions (82,306 patients) in 2009, 85,230 (80,238 patients) in 2010, and 83,797 (77,973 patients) in 2011. Most patients were men. Cases in which sex of the patient was not reported were excluded from the further analysis (7 cases in 2009, 7 cases in 2010 and 20 cases in 2011). All these admissions were treated solely at EDs.

### Patient age

**Fig. 1:**
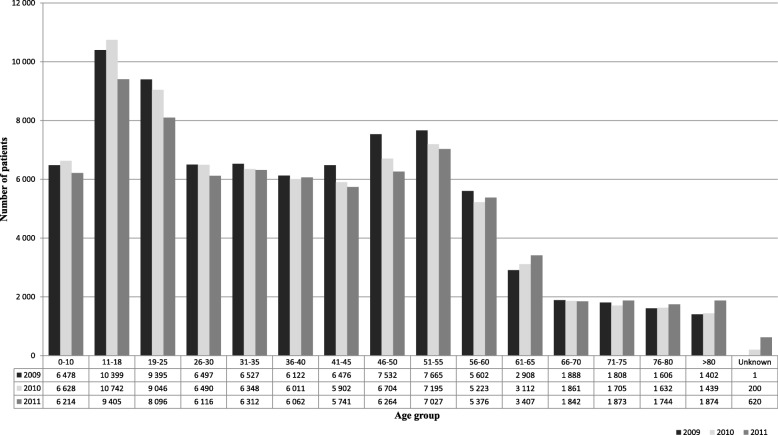
Age structure of patients admitted due to poisonings in 2009–2011

Distribution of patient age is shown in Fig. 1.

Patients 11–18 years of age (*n* = 30,546) were the largest group admitted between 2009 and 2011, followed by individuals aged 19–25 years (*n* = 26,537), 51–55 years (*n* = 21,887) and 46–50 years (*n* = 20,500). Cases where age was not reported were excluded from the age analysis.

### Causes of poisonings

Most common diagnoses reported in the analyzed period are presented in Table 2.

**Table 2:** Most common causes of admissions due to poisonings reported in the 2009–2011 period

ICD-10 code	Year			2009–2011 N (% of column)
	2009 N (% of column)	2010 N (% of column)	2011 N (% of column)	
T51.0 Ethanol	21,283 (24.9%)	19,471 (22.9%)	21,422 (25.6%)	62,176 (24.4%)
T51.9 Alcohol, unspecified or T51 partially coded	22,739 (26.6%)	17,975 (21.1%)	18,984 (22.7%)	59,698 (23.5%)
T58 Carbon monoxide	5064 (5.9%)	7151 (8.4%)	4964 (5.9%)	17,179 (6.8%)
T42.4 Benzodiazepines	3671 (4.3%)	3552 (4.2%)	3117 (3.7%)	10,340 (4.1%)
T45.5 Anticoagulants	2316 (2.7%)	2440 (2.9%)	3220 (3.8%)	7976 (3.1%)
T50.9 Other and unspecified drugs, medicaments and biological substances	1314 (1.5%)	1473 (1.7%)	1293 (1.5%)	4080 (1.6%)
T42.6 Other antiepileptic and sedative hypnotic drugs	1379 (1.6%)	1433 (1.7%)	1243 (1.5%)	4055 (1.6%)
T63.4 Venom of other arthropods	1023 (1.2%)	901 (1.1%)	1152 (1.4%)	3076 (1.2%)
T39.3 Other nonsteroidal anti-inflammatory drugs	966 (1.1%)	979 (1.2%)	982 (1.2%)	2927 (1.2%)
All admissions (T36-T65)	85,391 (100%)	85,223 (100%)	83,777 (100%)	254,391 (100%)

The most common diagnoses were alcohols. Altogether, intoxications with ethanol (T51.0), partially coded alcohol (T51) or unspecified alcohol (T51.9) represented nearly half of all recorded admissions due to poisonings (*n* = 121,874; 47.9%). There was a slight increase in the number of admissions reported as T51.0 and a decrease of poisonings with unspecified (T51.9) or partially coded alcohol (T51). Summarily the proportion of admissions due to the T51, T51.0 and T51.9 codes decreased from 51.5% in 2009 to 48.2% in 2011 (χ2 = 187.64; *p* < 0.01). There was an increase of admissions due to carbon monoxide poisonings in 2010 which amounted to 8.39% of all cases of poisonings, significantly higher than in 2009 and 2011 (5.93% and 5.92% respectively; χ2 = 546.26; *p* < 0.01). There was a decline in number of poisonings with benzodiazepines, which constituted 4.3% of all admissions in 2009 and 3.72% in 2011 (χ2 = 36.82; *p* < 0.01). There was a significant increase of admissions encoded as anticoagulants and antithrombotic drugs (T45.5) from 2.71% in 2009 to 3.84% in 2011 (χ2 = 170.83; *p* < 0.01).

The etiology of poisonings in the youngest groups of patients was different than in adults. The most common diagnoses in children and teenagers and the results of the statistical comparison between these groups are presented in Table 3.

**Table 3:** Most common poisonings in children and teenagers in the 2009–2011 period

ICD-10 code	0–10 years old (% of column)	11–18 years old (% of column)	χ^2^(p) 0–10 vs. 11–18^a^	Total
T51.0 Ethanol	442 (2.3%)	7814 (25.6%)	4646.06 (< 0.01)	8256 (16.6%)
T51.9 Alcohol, unspecified or T51 (partially coded)	325 (1.7%)	3007 (9.8%)	1263.79 (< 0.01)	3332 (6.7%)
T58 Carbon monoxide	2807 (14.5%)	2782 (9.1%)	350.04 (< 0.01)	5589 (11.2%)
T50.9 Other and unspecified drugs, medicaments and biological substances	908 (4.7%)	1073 (3.5%)	43.82 (< 0.01)	1981 (3.9%)
T63.4 Venom of other arthropods	1091 (5.6%)	478 (1.6%)	647.53 (< 0.01)	1569 (3.2%)
T39.3 Other nonsteroidal anti-inflammatory drugs	526 (2.7%)	880 (2.9%)	1.07 (0.301)	1406 (2.8%)
T42.4 Benzodiazepines	463 (2.4%)	910 (3.0%)	14.96 (< 0.01)	1373 (2.8%)
T48.3 Antitussives	61 (0.3%)	877 (2.9%)	418.53 (< 0.01)	938 (1.9%)
T42.6 Other antiepileptic and sedative hypnotic drugs	204 (1.1%)	662 (2.2%)	85.57 (< 0.01)	867 (1.7%)
T55 Soaps, detergents	575 (3.0%)	48 (0.2%)	762.61 (< 0.01)	623 (1.2%)
T39.1 4-Aminophenol derivatives	75 (0.4%)	536 (1.8%)	182.51 (< 0.01)	611 (1.2%)
T60 Pesticides	513 (2.7%)	65 (0.2%)	616.5 (< 0.01)	578 (1.2%)
All admissions (T36-T65)	19,316 (100%)	30,550 (100%)	–	49,866 (100%)

^a^Pearson’s chi-squared test

Like in the whole group, carbon monoxide was a common diagnosis in the two youngest age groups. Other common diagnoses in the 0–10 group were typical for accidental poisonings (venom of arthropods, detergents, pesticides). The T51, T51.0 and T51.9 codes were the most common causes of admissions of teenagers (*n* = 10,821; 35,4%), but the proportion was much lower than in adults (*n* = 110,286; 54.5%; χ2 = 1349.94; *p* < 0.01). Patients aged 11–18 were more often poisoned with nonsteroidal anti-inflammatory drugs (NSAIDs) than adults (2,9% vs 0,7%; χ2 = 1158.18; p < 0.01). Teenagers were less likely to overdose benzodiazepines than older patients (3% vs 4.4%; χ2 = 121.15; *p* < 0.01). There were 938 (1.9%) admissions of patients ≤18 years old due to poisonings with antitussives in the analyzed period. The number of these cases rose by 35%, from 256 (1.52%) in 2009 to 345 (2.21%) in 2011. In the analyzed period there was also a higher proportion of admissions due to venom of other arthropods in the 0–18 group than in adults: 1569 (3.2%) and 1507 (0.7%) cases respectively (χ2 = 1875.14; *p* < 0.01).

Etiology of poisonings differed significantly between the sexes. In the analyzed period there were 165,650 admissions of men and 88,741 admissions of women. Alcohols (all T51 codes) were the reason for 104,680 admissions in men (63.2%) and only 22,612 admissions in women (25.5%; χ2 = 32,873.6; *p* < 0.01). Poisonings with pharmaceuticals and other drugs (all codes within the T36-T50 range) were reported in 34,616 men (20.9%) and 45,238 women (51%; χ2 = 24,277.5; *p* < 0.01).

### Treatment at ED vs. hospitalization

In the whole analyzed period the total number of poisoned patients who were treated solely at EDs (without further hospitalization) was 127,575 (50.1%). The proportion of patients treated solely at EDs and hospitalized and the sex distribution in these groups are shown in Table 4.

**Table 4:** Number of admissions of acutely poisoned patients in the years 2009–2011, stratified by sex and place of treatment (Emergency Department vs. hospitalization)

Data category	Year									2009–2011		
	2009			2010			2011					
	M	F	Total	M	F	Total	M	F	Total	M	F	Total
All admissions	55,910	29,481	85,391	55,042	30,181	85,223	54,698	29,079	83,777	165,650	88,741	254,391
Cases treated solely in an Emergency Department (% of column)	31,969 (57.2%)	10,026 (34%)	41,995 (49.2%)	31,494 (57.2%)	10,152 (33.6%)	41,646 (48.9%)	33,326 (60,9%)	10,608 (36.5%)	43,934 (52.4%)	96,789 (58.4%)	30,786 (34.7%)	127,575 (50.1%)
Hospitalizations (% of column)	23,941 (42.8%)	19,455 (66%)	43,396 (50.8%)	23,548 (42.8%)	20,029 (66.4%)	43,577 (51.1%)	21,372 (39.1%)	18,471 (63.5%)	39,843 (47.6%)	68,861 (41.6%)	57,955 (65.3%)	126,816 (49.9%)

Women were generally more likely to be hospitalized and men were more frequently treated solely at EDs. The proportions of patients who were hospitalized in 2009, 2010 and 2011 were 66%, 66.4% and 63.5% for women and 42.8%, 42.8% and 39.1% for men (χ2 respectively 4146.56, 4337.91 and 4549.74; *p* < 0.01 for each analyzed year). The average age of patients treated solely at EDs was 40.3 ± 17.2 years and was significantly higher than the age of patients who were hospitalized (34.9 ± 22.3; *t* = 68.4101, *p* < 0.01). Alcohol poisonings (all T51 codes) constituted 70.8% of admissions which were treated solely at EDs compared with 25% of hospitalizations with this diagnosis in the 2009–2011 period (χ2 = 49,989.3; *p* < 0.01). Other common diagnoses for cases treated solely at EDs were carbon monoxide poisoning (5404 cases; 4.2%) and envenomation by arthropods (2106 cases; 1.7%). The most common diagnoses reported for hospitalized patients are presented in Table 5.

**Table 5:** Most common diagnoses of patients hospitalized due to poisonings in the 2009–2011 period

ICD-10 code	n (%)
T51.0 Ethanol	28,465 (22,4%)
T58 Carbon monoxide	12,333 (9.7%)
T42.4 Benzodiazepines	9497 (7.5%)
T45.5 Anticoagulants	7373 (5.8%)
T51.9 Alcohol, unspecified	3758 (3%)
T42.6 Other antiepileptic and sedative hypnotic drugs	3518 (2.8%)
T50.9 Other and unspecified drugs medicaments and biological substances	3004 (2.4%)
T39.3 Other nonsteroidal anti-inflammatory drugs	2693 (2.1%)
T43.0 Tricyclic and tetracyclic antidepressants	2134 (1.7%)
T43.2 Other and unspecified antidepressants	2063 (1.6%)
All hospitalizations (T36-T65)	126,816 (100%)

### Hospital units

In the analyzed period 94,514 cases of poisonings were hospitalized in internal medicine and pediatric departments (74.5% of all hospitalizations). From 2009 to 2011 28,996 (22.9%) patients were treated at RCCTs, there was an increase from 21% in 2009 to 25% in 2011. Only 3206 cases (2.5%) were treated in general Intensive Care Units (ICUs). The proportion of hospitalizations at ICUs increased from 1.8% in 2009 to 3.1% in 2011.

### Length of hospital stay

Mean duration of hospital stay due to poisonings remained constant throughout the analyzed period and amounted to 3 ± 11 days. We observed a slight decrease in the mean duration of hospital stays at RCCT: from 3.6 ± 5 days in 2009 to 3 ± 5 days in 2011.The proportion of hospitalizations longer than 10 days constituted less than 1% of the whole dataset.

### Mode of discharge

The data on the mode of hospital discharge after admissions due to poisoning in the studied period are presented in Table 6 .

**Table 6:** Mode of hospital discharge after poisonings in the 2009–2011 period

Mode of hospital discharge	Year			2009–2011 (% of column)
	2009 (% of column)	2010 (% of column)	2011 (% of column)	
Discharge without further referral	13,664 (31.5%)	13,458 (30.9%)	12,550 (31.5%)	39,672 (31.3%)
Referral to outpatient care	19,128 (44.1%)	19,165 (44%)	16,317 (41%)	54,610 (43.1%)
Referral to further hospitalization	4606 (10.6%)	5140 (11.8%)	5641 (14.2%)	15,387 (12.1%)
Discharge against medical advice	5506 (12.7%)	5137 (11.8%)	4452 (11.2%)	15,095 (12%)
Leaving hospital without discharge	0 (0%)	38 (0.1%)	250 (0.6%)	288 (0.2%)
Death	491 (1.1%)	580 (1.3%)	609 (1.5%)	1680 (1.3%)
Other/unspecified	1 (0.002%)	59 (0.1%)	24 (0.1%)	84 (0.1%)
Total number of hospitalizations	43,396 (100%)	43,577 (100%)	39,843 (100%)	126,816 (100%)

There was a decrease in the number of patients who were discharged against medical advice and concomitant increase in the number of individuals who self-willingly left the hospital without discharge.

### Mortality

The proportion of hospitalizations during which the patients died increased throughout the analyzed period, amounting to 1.1%, 1.3% and 1.5% in 2009, 2010 and 2011, respectively (χ2 = 196.89; *p* < 0.01). The proportion of patients who died during hospitalization at RCCT (109, 96 and 109 cases respectively) remained at a relatively stable level (around 1%). The increase of mortality in ICUs, from 14.4% (114 cases) in 2009 to 22.3% (271 cases) in 2011, is worth emphasizing (χ2 = 13.32; *p* < 0.01). The proportion of patients who died during hospitalization in other units (usually internal medicine and pediatric departments) remained at a relatively constant level (268, 270 and 229 cases respectively; around 0.8% of all reported cases). The most common diagnoses of reported fatal poisonings are presented in Table 7.

**Table 7:** Causes of fatal poisonings reported in the 2009–2011 period, stratified by the place of treatment

ICD-10 code	Number of fatal cases (% of column)			
	RCCTs	ICUs	other departments	all departments
T51.0 Ethanol	26 (8.3%)	79 (13.2%)	159 (20.7%)	264 (15.7%)
T45.5 Anticoagulants	0 (0%)	14 (2.3%)	134 (17.5%)	148 (8.8%)
T52.3 Glycols	56 (17.8%)	23 (3.8%)	32 (4.2%)	111 (6.6%)
T51.1 Methanol	34 (10.8%)	43 (7.2%)	23 (3.0%)	100 (6%)
T51.9 Alcohol, unspecified	5 (1.6%)	21 (3.5%)	69 (9%)	95 (5.7%)
T51 Alcohol, partially coded	1 (0.3%)	50 (8.3%)	37 (4.8%)	88 (5.2%)
T58 Carbon monoxide	18 (5,7%)	17 (2.8%)	22 (2.9%)	57 (3.4%)
T42.4 Benzodiazepines	16 (5.1%)	15 (2.5%)	25 (3.3%)	56 (3.3%)
T43.0 Tricyclic and tetracyclic antidepressants	12 (3.8%)	11 (1.8%)	9 (1.2%)	32 (1.9%)
T42.6 + T42.7 Other and unspecified antiepileptic and sedative-hypnotic drugs	6 (1.9%)	9 (1.5%)	16 (2.1%)	31 (1.8%)
T40.4 + T40.6 + T40.9 Other and unspecified narcotics and psychodysleptics (includes new psychoactive substances^a^)	9 (2.9%)	17 (2.8%)	5 (0.7%)	31 (1.8%)
T43.3 Phenothiazine antipsychotics and neuroleptics	2 (0.6%)	20 (3.3%)	7 (0.9%)	29 (1.7%)
T51.8 Other alcohols	17 (5.4%)	13 (2.2%)	11 (1.4%)	28 (1.7%)
T47.5 Anticoagulant antagonists, vitamin K and other coagulants	0 (0%)	5 (0.8%)	23 (3.0%)	28 (1.7%)
T46.1 Calcium channel blockers	12 (3.8%)	7 (1.2%)	6 (0.8%)	25 (1.5%)
T60.0 + T60.3 + T60.9 Pesticides	14 (4.5%)	10 (1.7%)	0 (0%)	24 (1.4%)
T38.3 Insulin and oral hypoglycemic (antidiabetic) drugs	4 (1.3%)	10 (1.7%)	7 (0.9%)	21 (1.3%)
T62.0 Mushroom ingestion	10 (3.2%)	7 (1.2%)	2 (0.3%)	19 (1.1%)
T43.8 Other psychotropic drugs	1 (0.3%)	10 (1.7%)	5 (0,7%)	16 (1.0%)
T39.1 4-Aminophenol derivatives (includes acetaminophen^a^)	7 (2.5%)	5 (0.8%)	3 (0.4%)	15 (0.9%)
T43.5 Other and unspecified antipsychotics and neuroleptics	1 (0.3%)	6 (1.0%)	8 (1.0%)	15 (0.9%)
T42.1 Iminostilbenes (includes carbamazepine^a^)	8 (2.5%)	5 (0.8%)	0 (0%)	13 (0.8%)
T45.1 Antineoplastic and immunosuppressive drugs	0 (0%)	0 (0%)	13 (1.7%)	13
T43.2 Other and unspecified antidepressants	3 (1.0%)	5 (0.8%)	3 (0.4%)	11 (0.7%)
T46.9 Other and unspecified agents primarily affecting the cardiovascular system	1 (0.3%)	5 (0.8%)	5 (0.7%)	11 (0.7%)
T44.7 Beta-adrenoreceptor antagonists	1 (0,3%)	5 (0.8%)	4 (0.5%)	10 (0.6%)
Other	50 (15.9%)	187 (31.2%)	139 (18.1%)	389 (23.2%)
All fatal cases (T36-T65)	314 (100%)	599 (100%)	767 (100%)	1680 (100%)

^a^authors’ comment

Diagnoses associated with ethanol (T51.0, T51 and T51.9 codes) were recorded in 447 (26.6%) fatal poisonings reported in the analyzed period. Most of these cases (92.8%) were reported by departments other than RCCTs, 150 (33.6%) by ICUs and 265 (59.3%) by other departments. Surprisingly, anticoagulants (T45.5) were the second most common diagnosis of fatal poisonings overall. No such cases were reported by RCCTs, 9.5% of these fatalities were reported by ICUs and 90,5% by other departments. There were also 28 fatal cases reported as anticoagulant antagonist poisonings, none of these cases were reported by RCCTs. The most common causes of fatal poisonings reported by RCCTs were glycols, methanol, ethanol, carbon monoxide and benzodiazepines. Poisonings with pesticides (all T60 codes) were the cause of 24 fatal cases and mushroom ingestions caused 19 deaths. Most of these cases were reported by RCCTs (58.3% and 52.6%, respectively). The codes typically used for new psychoactive substances (T40.4, T40.6 and T40.9) were reported in 31 fatal cases in the analyzed period. Most of these cases (54.8%) were reported by ICUs. In the analyzed period only 9 fatal cases of opioid poisonings (T40.1, T40.2 and T40.3 codes) were reported to the NHF by all hospital units in Poland.

## Discussion

### Number of admissions

The total number of admissions due to poisonings recorded in the 2009–2011 period amounted to 254,425, which corresponded to 84,808 admissions per year on average. However, if adjusted for cases not reported to the NHF due to lack of health insurance, the true number of admissions due to poisonings would be approximately 300,000 in the analyzed period or 100,000 per year. Importantly, we observed a small decreasing tendency in the annual number of admissions due to poisonings. Irrespective of the analyzed year, the proportions of patients who were admitted solely to EDs and those who required longer hospitalization remained roughly similar (around 50% each).

### Age and sex

The largest age group of patients who were admitted due to poisonings were teenagers, 11–18 years of age. Other authors reported similar age structure of poisoned patients [1, 2]. A relatively large proportion of middle-aged individuals (46–55 years of age) is worth emphasizing, as well as an increase in the number of poisoned subjects older than 80 years. The latter tendency most likely reflects the aging of Polish population and the changes in social and economic status of this age group [1–3, 16]. The increasing number of cases of poisonings in subjects older than 80 years is worrying, because of the increased mortality and longer hospital stay in this group [16].

Men constituted the vast majority of poisoned patients admitted during the analyzed period (average M/F ratio was 1.8). This observation is inconsistent with the data published by other authors according to which the majority of poisoned patients were women. This discrepancy can be explained by regional differences, e.g. status of health insurance or lack thereof and widely understood socioeconomic and cultural factors [2, 3, 17–19]. However we believe, that this difference is mostly due to two other factors. First, this study included not only cases treated in RCCTs, but also at EDs and men were much more frequently treated solely at EDs than women. Second, the main cause of poisonings in Poland in the reported period was ethanol and ethanol intoxication was much more common in men than in women (63.2% and 25.5% respectively). In our opinion these two factors were the main reason for the difference in sex distribution from other studies. Different sex distribution was seen for poisonings with pharmaceuticals and other drugs which were more frequent in women than in men (51% and 20.9% respectively).The available data were not sufficient to analyze the intent of poisoning (suicidal, accidental or recreational), but we believe, that the differences in diagnoses between men and women seen in our study are in line with previous findings that women were more likely to choose self-poisoning as a method of committing suicide than men [20].

### Etiology of poisonings

Etiology of poisonings varies widely between countries, depending on many factors including availability of pharmaceuticals and other toxic substances and also cultural, social and economic differences. In our study irrespective of the analyzed year, the most common cause of admissions due to poisoning were alcohols, especially ethanol. The incidence of ethanol intoxications is known to vary considerably depending on social acceptance to alcohol consumption or religious background [1, 16, 19, 21–23]. Ethanol abuse and dependence are a long-time problem in Poland. According to the 2014 WHO global status report on alcohol and health, the average consumption of alcohol in Poland (12,5 l of 100% ethanol per year) was slightly higher than the mean value for the WHO European Region (10,9 l). However, because a significant part of the Polish population are lifetime abstainers (15.8% men and 37.8% women), the mean amount consumed by the drinkers was much higher (24,2 l per year) [24]. The results of our study showed that ethanol was a significant cause of morbidity and mortality in the Polish population, especially among men. Alcohols were the leading cause of both admissions and deaths due to poisonings in the analyzed period. Ethanol dependence and abuse are complex problems, and because of that many national, local and non-government organization entities in Poland are involved in long-time anti-alcohol interventions. Production and sale of alcoholic products is strictly regulated and the criminal law is severe for alcohol-related offences. Encouragingly, in the analyzed period there was a decreasing tendency of admissions due to alcohols.

Pharmaceutical agents and other drugs (all codes within the T36-T50 range) turned out to be the second most frequent cause of admissions and the leading cause of hospitalizations. The number of such cases remained relatively stable throughout the analyzed period. Noticeably, there was an increase in the number of admissions and deaths due to poisonings with anticoagulants recorded as a primary diagnosis. Interpreting this phenomenon, is difficult without access to all clinical data. It is likely that many of these cases were not really poisonings and that the T45.5 diagnosis was reported to the NHF in cases of adverse effects of antithrombotic therapy or even underdosing of these agents. It is worth noting that none of the fatal cases of anticoagulant overdose were reported by RCCTs. An additional analysis of all clinical data would be of most importance, especially for the fatal cases. The authors believe that anticoagulant overdose should rarely be fatal given the treatment options available in Poland. Full cooperation of the NHF and the involved hospitals would be necessary to perform such an analysis.

Carbon monoxide was still a significant cause of morbidity and mortality in the analyzed period. There was a significant increase of both admissions and fatalities due to CO exposure in 2010. This was probably due to the fact that the winter months of 2010 (winters of 2009/2010 and 2010/2011) were significantly colder than usual in Poland, which resulted in a higher risk of household exposure to CO due to heating with coal ovens [25].

The large number of admissions due to arthropod venom reported may have been due to insect bites, but also may have been caused by inaccurate encoding of ED interventions due to tick *(Ixodoidea)* bites.

There was a decreasing number of admissions due to benzodiazepine poisonings. This may be due to their declining use since safer and less addictive substances become more popular, but longer observation is necessary to fully support this conclusion.

There were significant differences in etiology of poisonings in the two youngest age groups. Most common causes of admissions were also alcohols and carbon monoxide. The most common diagnoses in the 0–10 group were in line with accidental poisonings (carbon monoxide, venoms, detergents) and in the 11–18 group they were more likely due to intentional overdoses or recreational substance use (alcohols, benzodiazepines, antitussives). The proportion of alcohol intoxications in the 11–18 age group was much lower than in adults, but higher than reported in other European countries [26]. The proportion of admissions due to poisonings with nonsteroidal anti-inflammatory drugs in this group was higher and poisonings with benzodiazepines were less frequent than in adults. This is most likely due to limited access to prescription medications in the younger group. Higher percentage of admissions due to arthropod venom in children and adolescents than in adults most likely reflects the differences in outdoor activity. In the analyzed period there was an increasing number of poisonings with antitussives, which was most likely due to recreational abuse of over-the-counter medications (mostly dextromethorphan) by Polish teenagers [27, 28]. In contrast to reports from other countries, the numbers of admissions and fatal cases due to poisonings with pesticides and opioids were relatively low in the analyzed period in Poland [23, 29, 30].

### Hospitalizations

Mean duration of hospital stay of poisoned patients was relatively constant throughout the analyzed period. We observed a slight decrease in the mean duration of stays at RCCTs, which is consistent with the data reported by other authors according to whom the duration of relatively expensive hospitalization in specialized toxicology units was often shortened in favor of long-term treatment in other units, e.g. inpatient or outpatient psychiatric departments [4, 5]. The largest proportion of hospitalizations due to poisonings was reported by units other than RCCTs, such as internal medicine and pediatric departments. However, we observed an increase in the number of patients admitted to RCCTs, which implies that a growing number of poisoned patients were treated at specialized units. Another important finding of this study is a relatively high proportion of poisoned patients who had self-discharged home, against medical advice, as well as an increase in the number of patients who self-willingly left hospital without discharge. The largest proportion of patients who self-willingly left hospitals were treated at internal medicine and pediatric units, usually due to ethanol intoxications. Such patients are often reluctant to provide any health insurance data and their behavior markedly disorganizes functioning of a hospital unit and unnecessarily involves both their families and medical personnel [4, 5].

### Mortality

A systematic increase in the proportion of patients who died during hospital stay is worth emphasizing. According to other authors, mortality rates of patients hospitalized due to poisoning vary from 0.4 to 3% depending on a country and etiology of poisoning [1, 3–5, 15, 17, 22, 29, 31–34]. Importantly, the mortality was lowest in the RCCTs, which implies that such specialized units are optimally prepared for management of even the most severe cases. We observed an increase in the mortality of patients hospitalized in general ICUs. This phenomenon can be only partially explained by the selection of patients admitted to the ICUs based on severity, because the similar selection applies to the RCCTs which have their own Intensive Care Units. The limited dataset available from the NHF made it impossible to properly compare the mortality between RCCTs and ICUs in this study, but previous research which included only poisonings with methanol and ethylene glycol showed more than 2 times higher mortality rate in ICUs than in RCCTs in this group of patients after adjusting for severity [35, 36]. The increase of mortality in ICUs is especially alarming because previous studies demonstrated that these units least often from all the departments referred their patients for further specialized treatment at RCCTs [4, 5].

The analysis of fatal cases revealed a striking difference in reported diagnoses between RCCTs and other departments. The most common diagnoses reported by RCCTs were associated with typically severe poisonings (methanol, ethylene glycol, carbon monoxide, pesticides, mushrooms, cardiovascular agents) while other departments reported codes which in the authors’ opinion should not be associated with so high mortality (like anticoagulants and anticoagulant antagonists). This is very likely due to use of poisoning codes in cases of complications of therapeutic use of these agents or in cases where the real cause of death was the pathology for which the anticoagulants or anticoagulants were administered. The data available from the NHF did not allow us to explore this issue further.

### Limitations of the study

Unlike previous studies, our analysis included not only the data of patients treated at RCCTs, but also those admitted to other hospital units, which has not been done before in Poland. Nevertheless, we are well aware of all potential limitations of the presented results, inherent to the fact that they are derived from the data gathered primarily for accounting purposes. In the NFH system the primary diagnosis is strictly associated with billing and as such may be a subject to bias. In order to maximize the reimbursement, hospitals may report secondary diagnoses (from medical perspective) as primary ones. We believe that in some cases ICD-10 classification was misinterpreted, including not only poisonings, but also adverse effects of treatment, which properly should be reported using other codes (eg. K29, D50-D76, T88.7). Another difficulty was due to the incomplete coding, which in many cases did not include the part of the ICD-10 code following the decimal mark, especially for cases treated solely at EDs. This was especially true for alcohol poisonings. Many such cases were coded as unspecified alcohol poisoning (T51.9) or only partially coded as T51 without specifying the type of alcohol (part of the code after the decimal mark). Almost all the admissions which were coded this way were treated solely at EDs. The T51 code includes ethanol (T51.0), methanol (T51.1), 2-propanol (T51.2), fusel oil (T51.3), other alcohols (T51.8) and unspecified alcohols (T51.9). In our opinion the diagnosis of ethanol poisoning was by far the most likely one for T51 and T51.9 codes. Similarly the part of the code identifying the intent of poisoning (X-Y codes), which would allow to distinguish suicidal overdoses from accidental and recreational ones was not required by the NHF for billing and this data was very fragmentary.

Another limitation is that the ICD-10 classification does not differentiate between acute and chronic exposures. We believe that a vast majority of analyzed cases were acute poisonings, because in Poland chronic exposures are usually treated in an outpatient setting, but the available data were not sufficient to confirm this.

The analyzed data did not include all admissions due to poisonings in the 2009–2011 period, because not all admissions were reported by the hospitals to the NHF. The reports for the NHF did not include the data of admissions of individuals who lacked the NHF health insurance, were imprisoned or were residents of countries outside the European Union. This can be a source of a significant bias, because it applied to up to 20% of all patients treated at RCCTs (personal communications during annual meetings of heads of Polish RCCTs). The proportion of patients without NHF health insurance treated in other hospital units might have been be similar or even higher (especially at ED’s). We would like to be able to present more recent data, but so far we were not able to obtain the data for years 2012–2017 due to a change of NHF data sharing policy. Nevertheless, to the best of our knowledge, the hereby presented analysis is based on the largest of all datasets studied to date in Poland.

## Conclusions

In conclusion, the overall number of admissions due to poisonings decreased slightly during the study period, but poisonings remained a significant cause of morbidity and mortality in Poland and caused around 100,000 admissions per year. Alcohols were the main cause of admissions due to poisonings, accounting for about 50% of all cases. Carbon monoxide exposures were a significant cause of morbidity and mortality in the studied period in Poland. The increasing number of poisonings with antitussives in adolescents is worth noting. Men were more likely to be admitted due to poisonings than women. Around half of the cases of poisonings were managed solely at Emergency Departments. Men were more likely to be treated solely at Emergency Departments and women were more likely to be hospitalized. The increasing number of patients older than 80 years may lead to increased treatment costs and mortality. There was an increase of hospital mortality due to poisonings, most evidently in general Intensive Care Units. We believe that a national poison information and toxicovigilance system should be created in Poland, ideally allowing for near real-time monitoring of cases of poisonings. This system could be based on Regional Centers of Clinical Toxicology, which should exist in all Polish regions.

## Abbreviations

ED: Emergency Department
ICU: Intensive Care Unit
NHF: National Health Fund
RCCT: Regional Center of Clinical Toxicology
